# Interpretation of the effects of rumen acidosis on the gut microbiota and serum metabolites in calves based on 16S rDNA sequencing and non-target metabolomics

**DOI:** 10.3389/fcimb.2024.1427763

**Published:** 2024-06-28

**Authors:** Fanlin Wu, Peng Ji, Haochi Yang, Xiaopeng Zhu, Xiaohu Wu

**Affiliations:** ^1^ Key Laboratory of Veterinary Pharmaceutical Development, Ministry of Agricultural and Rural Affairs, Lanzhou Institute of Husbandry and Pharmaceutical Sciences of Chinese Academy of Agricultural Sciences, Lanzhou, China; ^2^ College of Veterinary Medicine, Gansu Agricultural University, Lanzhou, China; ^3^ Zhangye Wanhe Grass Livestock Industry Science and Technology Development Co., Ltd, Zhangye, China

**Keywords:** calves, rumen acidosis, gut microbiota, serum metabolites, correlation analysis

## Abstract

**Introduction:**

Rumen acidosis is one of the most common diseases in beef cattle. It severely affects the normal development of calves and poses a significant threat to the farming industry. However, the influence of rumen acidosis on the gut microbiota and serum metabolites of calves is currently unclear.

**Objective:**

The aim of this study is to investigate the changes in the gut microbiota and serum metabolites in calves after rumen acidosis and analyse the correlation.

**Methods:**

Eight calves were selected as the rumen acidosis group, and eight health calves were selected as the healthy group. The faecal gut microbiota and serum metabolites of calves were detected respectively using 16S rDNA high-throughput sequencing and non-target metabolomics. The correlation between gut microbiota and serum metabolites was analyzed by Spearman correlation analysis.

**Results:**

Differential analysis of the diversity and composition of gut microbiota between eight male healthy (Health) and eight male rumen acidosis (Disease) calves revealed that rumen acidosis increased the abundance of the gut microbiota in calves. At the phylum level, compared to the Healthy group, the relative abundance of Proteobacteria in the Disease group significantly decreased (*P*<0.05), while the relative abundance of Desulfobacterota significantly increased in the Disease group (*P*<0.05). At the genus level, compared to the Disease group, the relative abundance of *Alloprevotella*, *Muribaculaceae*, *Succinivibrio*, *Prevotella*, *Agathobacter* and *Parabacteroides* significantly increased in the Healthy group (*P*<0.05), while the relative abundance of *Christensenellaceae_R-7* and *Monoglobus* significantly decreased in the Healthy group (*P*<0.05). Differential analysis results showed the Healthy group had 23 genera with higher abundance, while the Disease group had 47 genera with higher abundance. Serum metabolomics results revealed the differential metabolites associated with rumen acidosis, including nicotinamide, niacin, L-glutamic acid and carnosine, were mainly enriched in the nicotinate and nicotinamide pathway and the histidine pathway.

**Conclusion:**

The occurrence of rumen acidosis can induce changes in the gut microbiota of calves, with a significant increase of the *Christensenellaceae_R-7* genus and a significant decrease of *Prevotella* and *Succinivibrio* genera. In addition, the occurrence of rumen acidosis can also induce changes in serum metabolites including niacin, niacinamide, L-glutamine, and carnosine, which may serve as the diagnostic biomarkers of rumen acidosis of calves.

## 1 Introduction

Rumen acidosis is an important disease of the digestive system, and it seriously affects the health of cattle (Trevisi et al., 2018). In the clinical practice, it usually presents as the subacute symptoms with the typical signs of anorexia, depression, eyes sunken and muscle tremors. The cattle being severely affected may exhibit recumbency or opisthotonus, groan and excrete yellow-brown watery feces. From the above clinical symptoms, it can be seen that the rumen acidosis can lead to several intestinal diseases. The gut microbiota is closely related to the healthy status, nutrient metabolism, immune function, and the onset and development of disease (Niederwerder, 2018). The gut microbiota is involved in the regulation of various metabolic pathways in the host, forming interactions such as the host-microbe metabolic axis, the host-microbe signalling axis and the immune-inflammatory axis, which are closely related to the multiple organs in the body, including the gut, liver, muscle and brain (Welch et al., 2022). For example, when high-concentrate feeding is used to induce rumen acidosis, a large amount of undigested feed enters the hindgut, leading to fermentation in the hindgut and lowering the pH of the feces to around 6.42 (Gressley et al., 2011). High-throughput sequencing of the V1-V3 variable regions in bacteria revealed significant changes in the microbial community in the feces. Rumen acidosis causes large amounts of rumen fermentable substrates to enter the hindgut, producing large amounts of volatile fatty acids and lactic acid, which damage the intestinal epithelial cells. By altering the structure of the microbial community in the hindgut, diarrhea is induced, severely affecting the animal health and production (Mao et al., 2012). Rumen acidosis can activate the innate immune response in cattle, increasing the levels of serum amyloid A, lipopolysaccharide-binding protein and haptoglobin (Khafipour et al., 2009). This activation of the systemic immune response can be used as a diagnostic marker for rumen acidosis and also indicate the association between rumen acidosis and systemic health disorders (Zebeli and Metzler-Zebeli, 2012). In addition, further research has shown that rumen acidosis could also cause metabolic diseases such as ketosis and hyperlactatemia in dairy cows (Aditya et al., 2018; Tang et al., 2024). Currently, there is relatively little research on the changes in the fecal microbiota in calves with rumen acidosis. To analyze the changes in the gut microbiota structure and serum metabolites of rumen acidosis in calves, the microbial 16S rDNA V3-V4 region sequences in the feces and the serum metabolites of the healthy and rumen acidosis in calves were determined used the Illumina Miseq sequencing platform and LC-MS/MS in this study. To further elucidate the pathogenesis of rumen acidosis of calves, community structure diversity, differential metabolites screening, metabolic pathway analysis and the correlation analysis were performed.

## 2 Materials and methods

### 2.1 Main instruments and reagents

Ultra-performance liquid chromatograph (Waters 2D UPLC, Waters, USA); high-resolution mass spectrometer (Q-Exactive, Thermo Fisher Scientific, USA); chromatographic column: ACQUITY UPLC BEH C18 (1.7 μm, 2.1*100 mm, Waters, USA); low-temperature high-speed centrifuge (Centrifuge 5430, Eppendorf); vortex mixer (QL-901, Qilinbell Instrument Manufacturing Co., Ltd., China); water purification system (Milli-Q Integral, Millipore Corporation, USA); freeze vacuum concentrator (Maxi Vacbeta, GENE COMPANY); internal standards: L-leucine-d3, L-phenylalanine (13C-9.99%), L-tryptophan-d5, progesterone-2,3,4-13C3; methanol (A454-4) and acetonitrile (A996-4) were MS grade (Thermo Fisher Scientific, USA); formic acid ammonia (17843-250G, Honeywell Fluka, USA), formic acid (50144-50 mL, DIMKA, USA) and the water provided by the water purification system.

### 2.2 Experimental animals

The animals were provided by Zhangye Wanhe Grass Livestock Industry Science and Technology Development Co., Ltd. According to the performance and clinical signs (Oetzel, 2017), eight male calves were selected as the rumen acidosis group (Disease), and eight male healthy calves (with the similar age and weight to the rumen acidosis group) were selected as the healthy group (Health). Calves in the two groups were reared in the same feeding environment. The feed was supplied by Zhangye Wanhe Grass Livestock Industry Science and Technology Development Co., Ltd. The concentrated feed consists of corn, corn germ meal, soybean meal, distillers grains, baking soda and premix (**Table 1**). Written informed consent was obtained from the owners for the participation of their animals in this study.

**Table 1:** The composition of the basal diet.

Composition	Content(%)
Corn	52.00
Corn germ meal	12.00
Soybean meal	23.00
Distillers grains	8.00
Baking soda	1.00
Premix	4.00

### 2.3 Sample collection

#### 2.3.1 Feces sample collection

5 g feces samples were collected from each calf in the healthy group and the rumen acidosis group. Then the samples were placed in cryovials, and then immediately freezed in liquid nitrogen for the subsequent analysis.

#### 2.3.2 Serum sample collection

Blood samples were collected from the jugular vein of calves in the healthy and rumen acidosis groups. Then the serum was separated (centrifuged at 3500 rpm for 15 min) and stored at -80°C.

### 2.4 Metabolite extraction

The samples were thawed slowly at 4°C. Then, 100 μL of the sample was transferred to a 96-well plate and 300 μL of pre-cooled extraction solution (methanol:acetonitrile=2:1, v:v) along with 10 μL of internal standard were added. The mixture was made vortex for 1 min, and then centrifuged at 4°C, 4000 rpm for 20 min. After centrifugation, 300 μL of the supernatant was transferred to a freeze vacuum concentrator and dried. 150 μL complex solution (methanol:H_2_O=1:1, v:v) was added for re-dissolution. The solution was made vortex for 1 min, then centrifuged at 4°and 4000 rpm for 30 min. After that, the supernatant was transferred to the sample vials. To ensure the quality control, 10 μL of the supernatant from each sample was mixed to create the QC samples. These QC samples were used to evaluate the repeatability and stability of the LC-MS/MS analysis process.

### 2.5 LC-MS/MS analysis

Waters 2D UPLC (Waters, USA) conjunction with the Q Exactive high-resolution mass spectrometer (Thermo Fisher Scientific, USA) were utilized for metabolite separation and detection.

#### 2.5.1 Chromatographic conditions

BEH C18 column (1.7 μm, 2.1*100 mm, Waters, USA) was used. In the positive ion mode, the mobile phase consisted of 0.1% formic acid in aqueous solution (A) and 0.1% formic acid in methanol (B). In the negative ion mode, the mobile phase consisted of 10 mM formic acid ammonium solution (A) and 10 mM formic acid ammonium solution in 95% methanol (B). The gradient elution conditions were as follows: 0-1 min, 2% B; 1-9 min, 2%-98% B; 9-12 min, 98% B; 12-12.1 min, 98% B-2% B; 12.1-15 min, 2% B. The flow rate was 0.35 mL/min, the column temperature was 45°C, and the injection volume was 5 μL.

#### 2.5.2 Mass spectrometry conditions

Q-Exactive mass spectrometer was used for the primary and secondary mass spectrometry data acquisition. The mass scanning range was 70 - 1050 m/z, with a primary resolution of 70,000, AGC of 3e^6^, and the maximum injection time of 100 ms. The top 3 ions were selected for fragmentation based on precursor ion intensity, and secondary information was collected. The secondary resolution was set at 17,500, AGC was set at 1e^5^, and the maximum injection time was set at 50 ms. The fragmentation energy was set at 20, 40, and 60 eV. The ion source (ESI) parameters were set as follows: sheath gas flow rate at 40, auxiliary gas flow rate at 10, spray voltage at 3.80 KV in the positive ion mode and 3.20 KV in the negative ion mode, ion transfer tube temperature at 320°C, and auxiliary gas heater temperature at 350°C.

### 2.6 OTUs clustering result statistics

The software USEARCH (v7.0.1090) was used to cluster the assembled Tags into OTUs. The main process is as follows:

a. UPARSE was used to cluster at 97% similarity, obtaining representative sequences of OTUs.
b. UCHIME (v4.2.40) was used to remove chimeras from the representative sequences of OTUs. For 16S and ITS, pre-existing chimera databases were used for comparison and removal. The chimera databases used were gold database (v20110519) for 16S and UNITE (v20140703) for ITS, selected based on sequencing regions.
c. The usearch global method was used to align all Tags to the representative sequences of OTUs, obtaining the OTUs abundance table for each sample.

The DADA2 (Divisive Amplicon Denoising Algorithm) method in the software QIIME2 was used to denoise the data and obtain Amplicon Sequence Variants (ASVs), which were sequences with 100% similarity. Then, a feature table (Feature, a general term for ASV/ASV, etc.) was obtained. The main process was as follows:

a. Qiime tools were used to import filtered paired-end sequences.
b. Qiime DADA2 denoise-paired command was used to build the feature table based on the DADA2 method.
c. Qiime tools were used to export to convert the feature table into a format that can be directly viewed.

### 2.7 Data processing

The data were expressed as “mean ± standard deviation” and the statistical analysis was performed by GraphPad Prism 7.0. *P<0.05* was considered as statistically significant.

## 3 Results

### 3.1 Effects of rumen acidosis in calves on the gut microbiota

#### 3.1.1 Effects of rumen acidosis in calves on the OTUs of the gut microbiota

**Figure 1:**
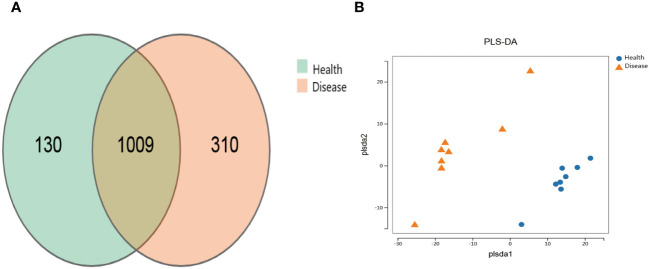
Effect on the distribution of gut microbiota OTUs of rumen acidosis in calves. Health, the healthy group; Disease, the rumen acidosis group. **(A)** represents the OTUs of the gut microbiota of the healthy group and the rumen acidosis group. **(B)** represents the PLS-DA analysis results of the healthy group and the rumen acidosis group.

The healthy group (Health) had 1139 operational taxonomic units (OTUs), while the rumen acidosis group (Disease) had 1319 OTUs, with 1009 OTUs shared between the two groups (**Figure 1A**). The PLS-DA analysis results clearly demonstrate a separation between the two groups (**Figure 1B**).

#### 3.1.2 Effects of rumen acidosis in calves on the gut microbiota diversity and richness

**Figure 2:**
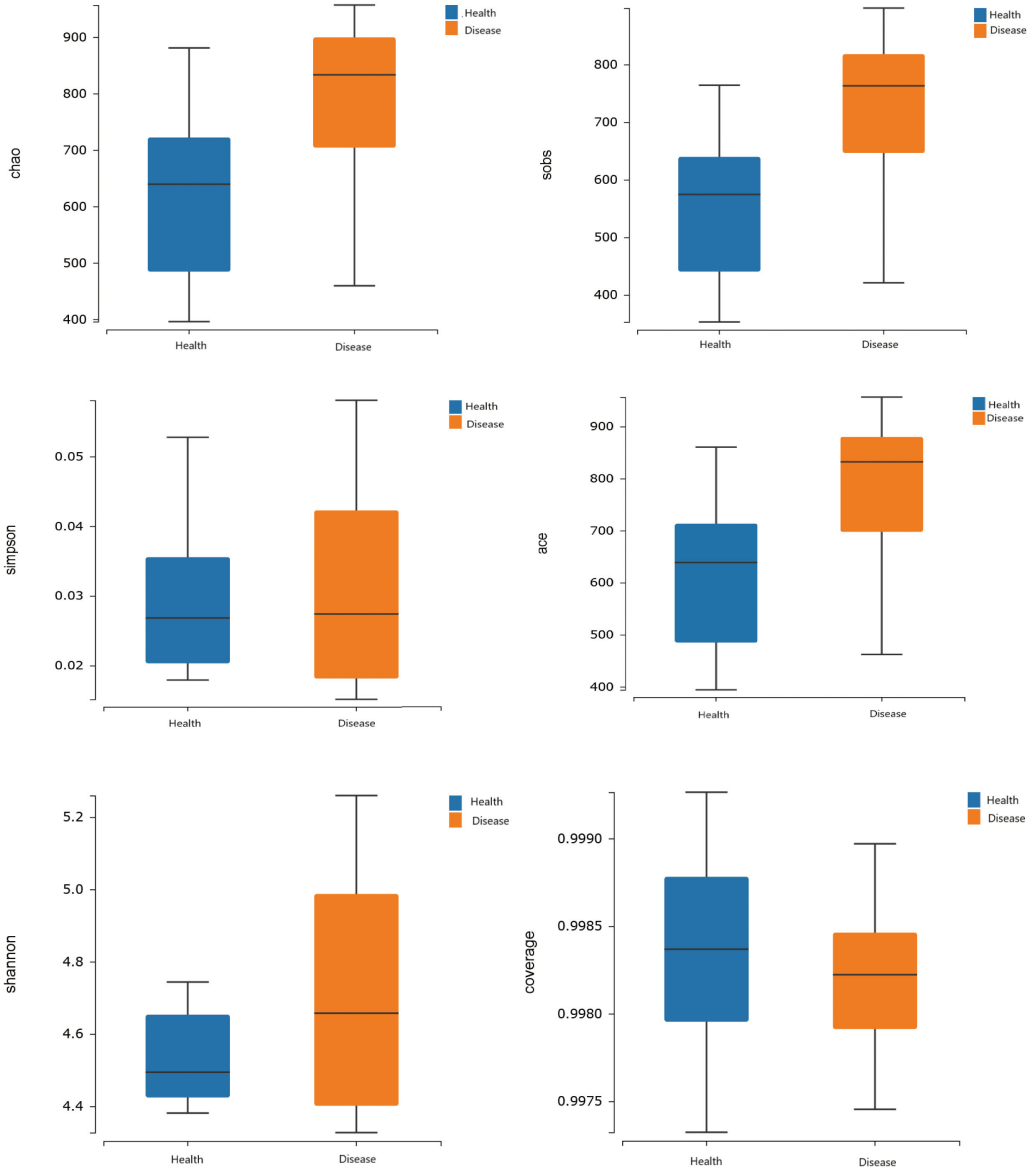
Effects of rumen acidosis on the α diversity of the gut microbiota in calves.

The analysis results of alpha diversity clearly demonstrated that there were differences in the richness of gut microbiota between the two groups. The rumen acidosis group (Disease) had significantly higher sobs, chao, and ace indices than the healthy group (Health) (*P<0.05*) (**Figure 2**). It strongly suggested that the rumen acidosis had a direct impact on the richness of the gut microbiota in the calves.

#### 3.1.3 Effects of rumen acidosis on the composition of the gut microbiota in the calves

**Figure 3:**
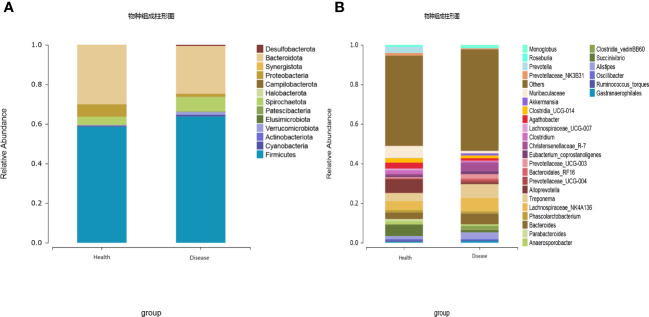
Effect of rumen acidosis on the gut microbiota of calves **(A)** represents phylum level; **(B)** represents genus level.

The gut microbiota of calves was primarily composed of Firmicutes, Bacteroidota, Spirochaetota, Proteobacteria, Cyanobacteria, Verrucomicrobiota, Desulfobacterota, Patescibacteria, Actinobacteriota, and Elusimicrobiota, with Firmicutes and Bacteroidota being the dominant phyla. The results indicated that the relative abundance of Proteobacteria was significantly lower in the rumen acidosis group (Disease) compared to the healthy group (Health) (*P<0.05*). Moreover, the rumen acidosis group (Disease) showed a significantly higher relative abundance of Desulfobacterota than the healthy group (Health) (*P<0.05*) (**Figure 3A**).

**Figure 4:**
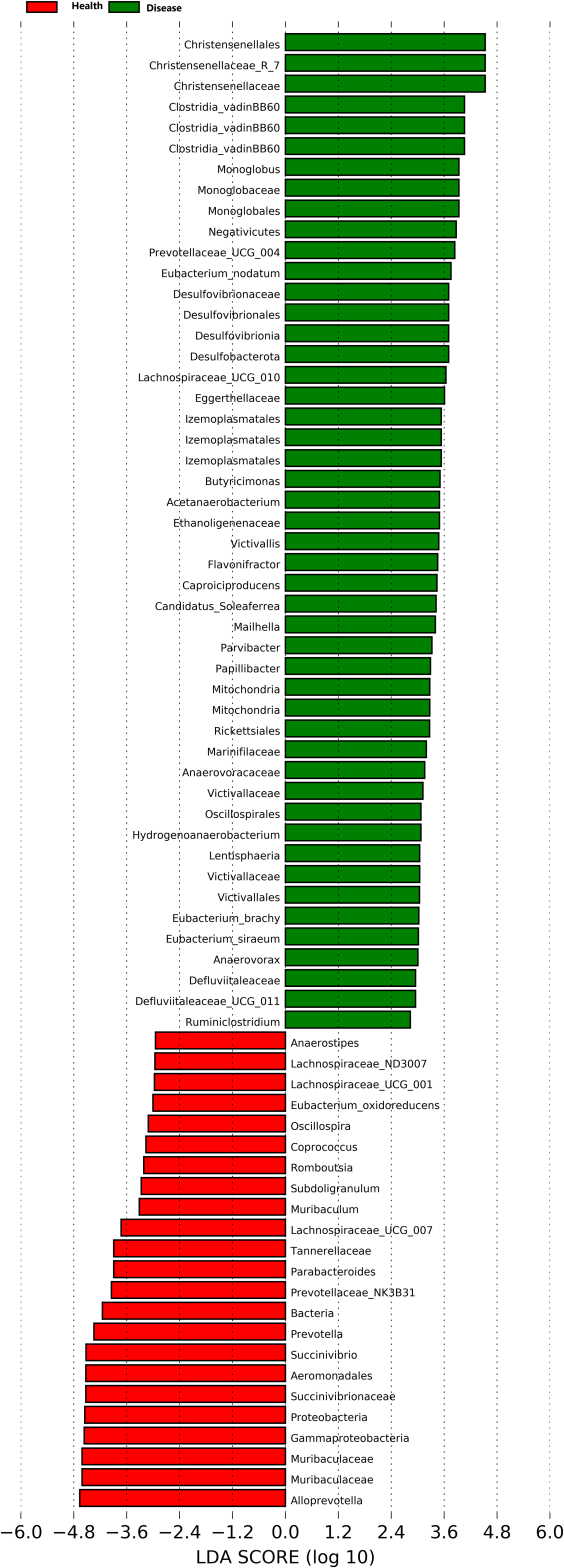
LDA analysis of rumen acidosis on the gut microbiota of calves.

At the genus level, twenty-eight dominant bacterial genera were identified, including *Alloprevotella*, *Muribaculaceae*, *Succinivibrio*, *Lachnospiraceae_NK4A136*, *Treponema*, *Bacteroides*, *Prevotella*, *Agathobacter*, *Clostridia_UCG-014*, *Clostridium*, *Alistipes*, *Anaerosporobacter*, *Prevotellaceae_NK3B31*, *Phascolarctobacterium*, *Parabacteroides*, *Eubacterium coprostanoligenes*, *Lachnospiraceae UCG-007*, *Christensenellaceae R-7*, *Oscillibacter*, *Ruminococcus torques*, *Roseburia*, *Gastranaerophilales*, *Prevotellaceae UCG-003*, *Clostridia vadinBB60*, *Monoglobus*, *Bacteroidales RF16*, *Akkermansia*, and *Prevotellaceae UCG-004*. The results indicated that the healthy group (Health) had a significantly higher relative abundance of *Alloprevotella*, *Muribaculaceae*, *Succinivibrio*, *Prevotella*, *Agathobacter*, and *Parabacteroides* compared to the rumen acidosis group (Disease) (*P<0.05*). Furthermore, the healthy group (Health) had a significantly lower relative abundance of *Christensenellaceae_R-7* and *Monoglobus* (*P<0.05*) (**Figure 3B**). When setting a predefined LDA value of >2.4, the results revealed that the healthy group (Health) had 23 genera with higher abundance, whereas the rumen acidosis group (Disease) had 47 genera with higher abundance at different taxonomic levels. This is demonstrated in **Figure 4**.

### 3.2 Effects of rumen acidosis in calves on the serum metabolites

#### 3.2.1 Instrument stability and total ion chromatograms in the positive and negative ion modes

**Figure 5:**
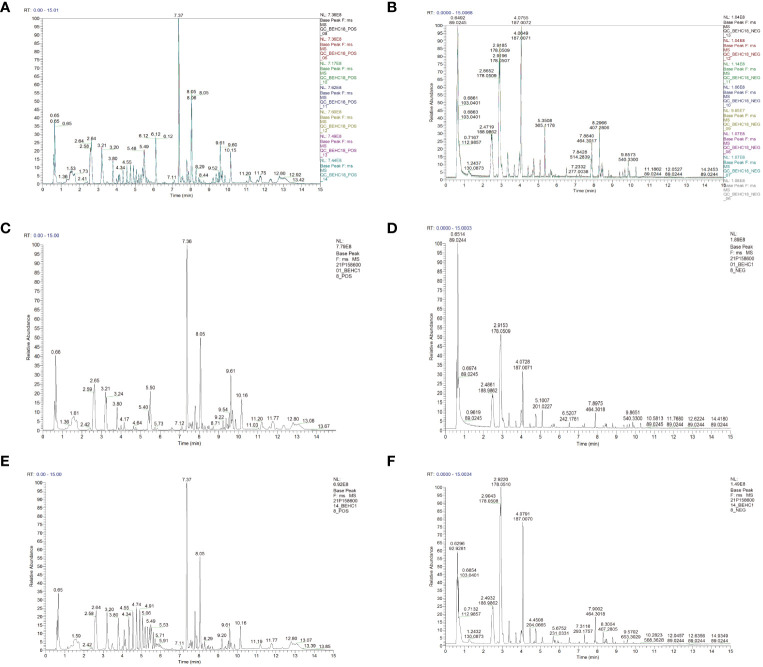
The instrumental stability and total ion current chromatogram of calves serum LC-MS/MS in the positive and negative ion modes **(A)** (positive ion); **(B)** (negative ion); **(C)** Health (positive ion); **(D)** Health (negative ion); **(E)** Disease (positive ion); **(F)** Disease (negative ion).

The total ion chromatograms of the six QC samples were consistent and reliable. The excellent stability of the instrument in both positive and negative ion modes was demonstrated in **Figures 5A, B**, and the reproducibility of the metabolomics method based on LC-MS/MS was evidenced in this experiment. The base peak chromatograms (BPC) of all the QC samples overlapped well, with only minor fluctuations in retention time and peak response intensity. This confirmed that the instrument was in a good condition and the signal stability was maintained throughout the sample analysis process.

#### 3.2.2 PCA analysis

**Figure 6:**
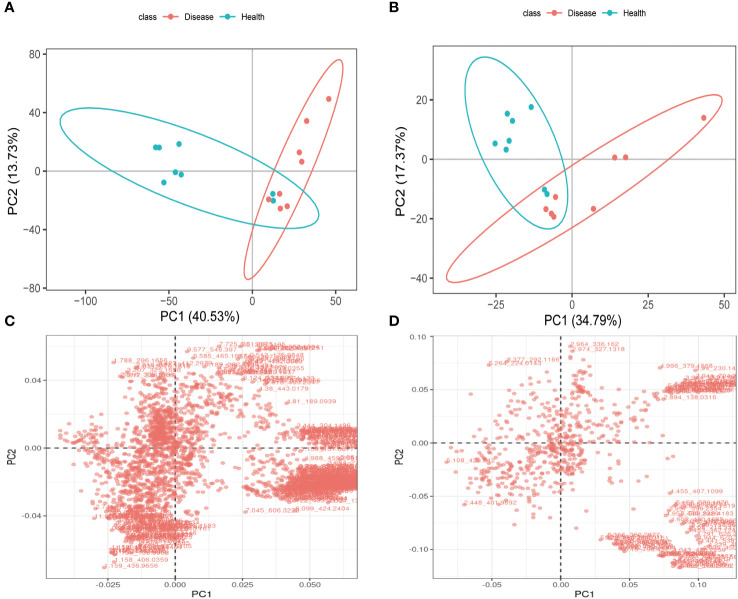
The PCA analysis and differential metabolites in each group of calves serum under LC-MS/MS positive ion **(A, C)** and negative ion **(B, D)** models.

PCA analysis results clearly demonstrated a distinct separation between the health and disease groups in terms of the metabolites detected in positive and negative ion modes. These findings strongly suggested that the rumen acidosis severely disrupted the normal physiological metabolism of calves, resulting in the significant changes in endogenous physiological metabolites (**Figures 6A, B**). In both positive and negative ion modes, the PCA score charts visually reflected the contribution of all metabolites to the intergroup differences. The outliers that were further away from the other metabolites had a greater contribution to the intergroup differences and were more likely to be the differential metabolites of rumen acidosis in calves, as shown in **Figures 6C, D**. The data confidently demonstrated a greater number of metabolites were detected in the negative ion mode.

#### 3.2.3 PLS-DA analysis of serum metabolites in the positive and negative ion modes

**Figure 7:**
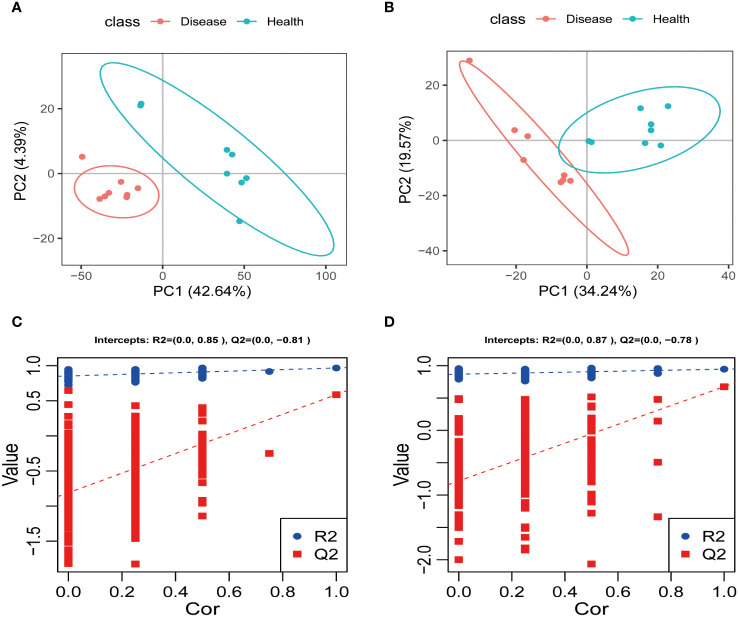
The PLS-DA analysis and scores of each group in the positive **(A, C)** and negative ion **(B, D)** models.

The positive and negative ion modes were used to conduct PLS-DA analysis on the serum metabolites of the healthy and disease groups. The sample points of the two groups were distinctly separated in both ion modes, and each group’s samples showed a tendency to be closer to their respective groups to varying degrees. These findings strongly suggested that rumen acidosis significantly impacted the normal metabolism of calves serum (**Figures 7A, B**). The PLS-DA model’s effectiveness was evaluated using R^2^Y and Q^2^. The study demonstrated that the PLS-DA score plot performed well and had high predictive ability, as evidenced by R^2^Y and Q^2^ values of 0.85 and 0.81, respectively. Additionally, the permutation test indicated that the model was not overfitting, with a Q^2^ intercept value less than 0 (**Figures 7C, D**).

#### 3.2.4 Screening of differential metabolites

The differential metabolites are shown in **Table 2** (in the **Supplementary materials**). A total of 139 differential metabolites were identified in the serum of calves in the positive ion mode using the PLS-DA model (VIP>1, Ratio>1.2 or Ratio<0.83, *P<0.05*), with 59 upregulated and 80 downregulated metabolites. In the negative ion mode, 39 differential metabolites were identified in the serum of calves, including 30 upregulated and 9 downregulated metabolites.

#### 3.2.5 Metabolic pathway enrichment analysis

**Figure 8:**
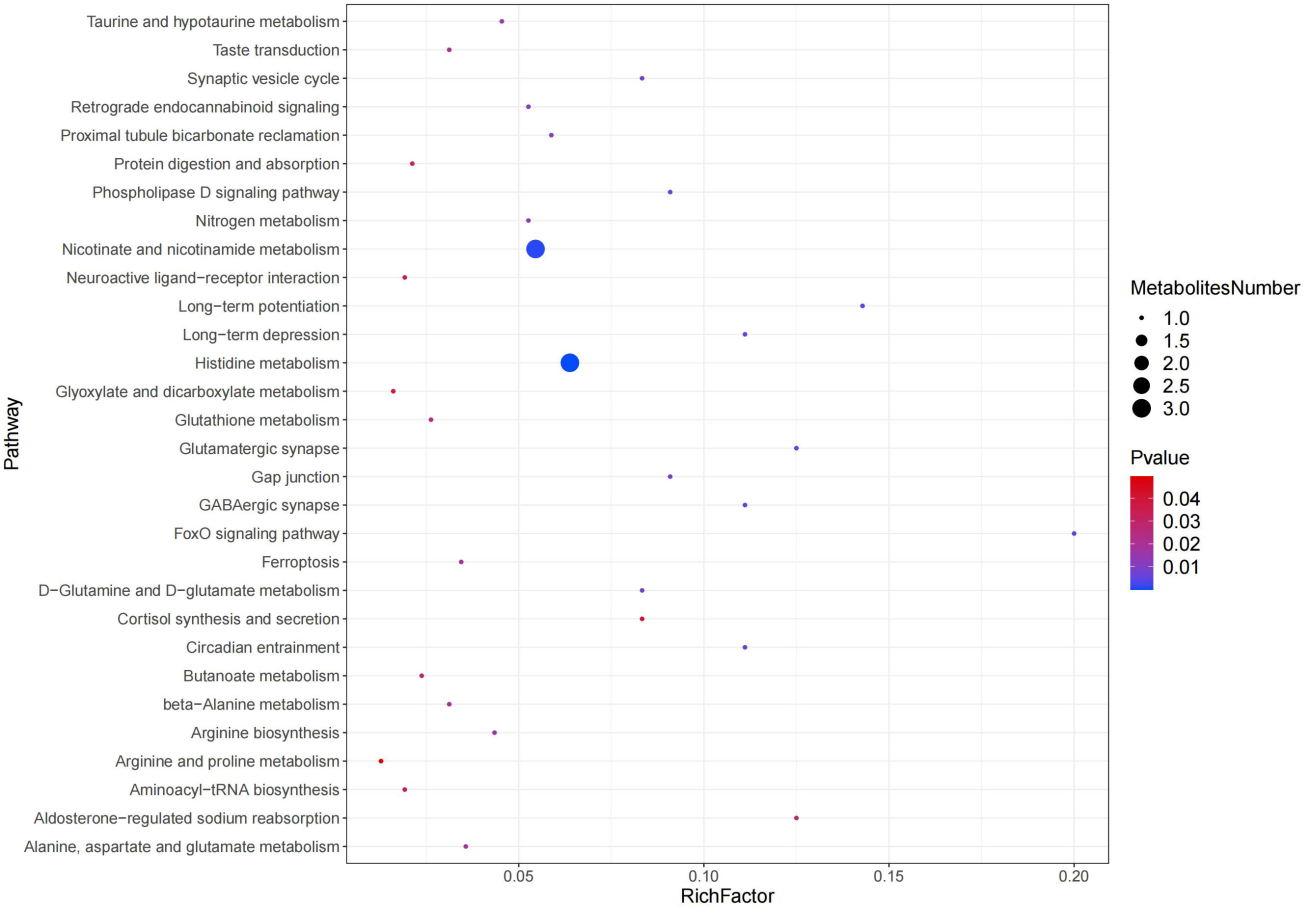
Pathway enrichment analysis results in the positive and negative ion modes.

The metabolite pathways with *P<0.05* were confidently selected as the most relevant to rumen acidosis in calves. In the positive ion mode, three metabolic pathways were decisively chosen, including Nicotinate and nicotinamide metabolism, Aldosterone-regulated sodium reabsorption, and Cortisol synthesis and secretion. 27 metabolic pathways were confidently identified in the negative ion mode, including Arginine and Proline metabolism, Glyoxylate and Dicarboxylate metabolism, Neuroactive Ligand-Receptor Interaction, Aminoacyl-tRNA Biosynthesis, Protein Digestion and Absorption, Histidine metabolism, Butanoate metabolism, Glutathione metabolism, Taste Transduction, Beta-Alanine metabolism, Alanine, Aspartate and Glutamate metabolism, Ferroptosis, Arginine biosynthesis, Taurine and hypotaurine metabolism, Retrograde endocannabinoid signaling, Nitrogen metabolism, Proximal tubule bicarbonate reclamation, D-Glutamine and D-glutamate metabolism, Synaptic vesicle cycle, Gap junction, Phospholipase D signaling pathway, Long-term depression, Circadian entrainment, GABAergic synapse, Glutamatergic synapse, Long-term potentiation, and FoxO signaling pathway (**Figure 8**).

### 3.3 Correlation analysis between gut microbiota and serum metabolites

**Figure 9:**
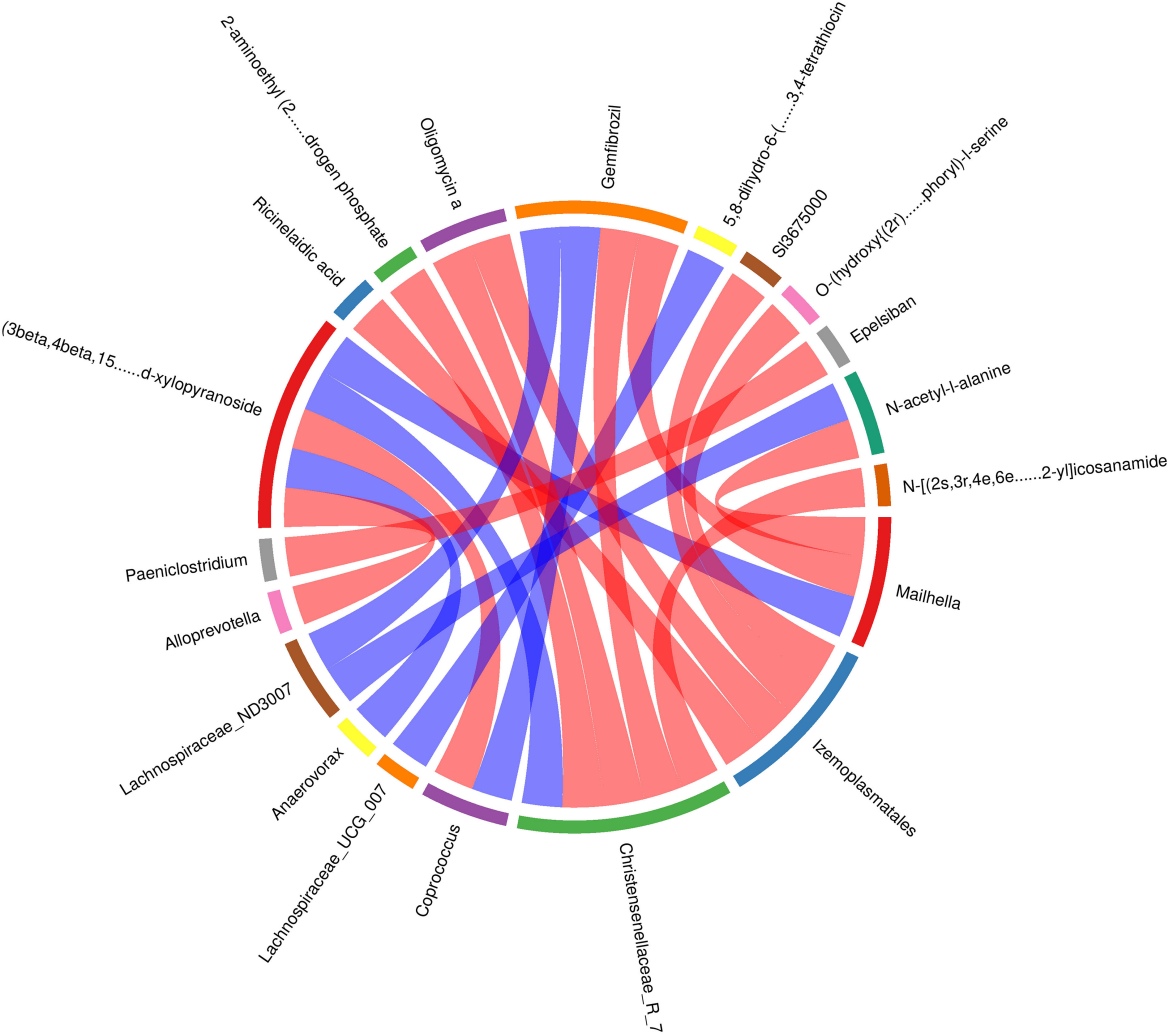
Correlation analysis of gut microbiota and metabolites in calves with rumen acidosis.

The spearman correlation analysis showed a negative correlation (r=0.89; *P<0.01*) between the relative concentration of (3β, 4β, 15α, 16β, 25s)-4, 8, 15, 16, 26-pentahydroxycholest-5-en-3-yl β-d-xylopyranoside and the *Alloprevotella* genus, and a positive correlation (r=0.90; *P<0.01*) with the *Anaerovorax* genus. The concentration of 2-aminoethyl (2r)-2-hydroxy-3-[(1z,12z)-1,12-nonadecadien-1-yloxy] propyl hydrogen phosphate was found to be negatively correlated with the presence of the *Christensenellaceae_R-7* genus (r=-0.94, *P<0.01*). Similarly, the concentration of Oligomycin a was also found to be negatively correlated with the presence of the *Christensenellaceae_R-7* genus (r=-0.92, *P<0.01*). The concentration of (3β,4β,15α,16β,25s)-4,8,15,16,26-pentahydroxycholest-5-en-3-yl β-d-xylopyranoside showed a positive correlation with the *Christensenellaceae_R-7* genus (r = 0.92, *P < 0.01*), while the concentration of Gemfibrozil exhibited a negative correlation with the same genus (r=-0.89, *P < 0.01*). The concentration of N - [(2s,3r,4e,6e)-1,3-dihydroxy-4,6-tetradecadien-2-yl] icosanamide showed a negative correlation with the presence of the *Christensenellaceae_R-7* genus (r=-0.89, *P < 0.01*).The concentration of (3β, 4β, 15α, 16β, 25s) - 4, 8, 15, 16, 26 - pentahydroxycholest-5-en-3-yl β-d-xylopyranoside showed a negative correlation with the presence of the *Coprococcus* genus (r=-0.93, *P<0.01*). Conversely, the concentration of Gemfibrozil showed a positive correlation with the presence of the *Coprococcus* genus (r=0.93, *P<0.01*). Furthermore, the concentration of Ricinelaidic acid exhibited a negative correlation with the presence of the *Izemoplasmatales* genus(r=-0.94, *P<0.01*). The negative correlation between the concentration of Oligomycin a and the *Izemoplasmatales* genus (r=-0.93, *P<0.01*), as well as the concentration of Sl3675000 and O-(hydroxy{(2r)-2-hydroxy-3-[(2-methoxyicosyl)oxy]propoxy}phosphoryl)-l-serine with the *Izemoplasmatales* genus (r=-0.90, *P<0.01*), demonstrates a clear relationship between these variables. Conversely, the concentration of Gemfibrozil and N-acetyl-l-alanine exhibited significant positive correlation with the *Lachnospiraceae_ND3007* genus (r=0.90 and 0.89, *P<0.01*) respectively. The concentration of 5,8-dihydro-6-(4-methyl-3-penten-1-yl)-1,2,3,4-tetration was strongly correlated with the presence of the *Lachnospiraceae_UCG_007* genus (r=0.90, *P<0.01*). Similarly, the concentration of (3β,4β,15α,16β,25s) - 4,8,15,16,26-pentahydroxycholest-5-en-3-yl β- d - xylopyranoside was highly correlated with the presence of the *Mailhella* genus (r=0.96, *P<0.01*). The concentration of Gemfibrozil and N-acetyl-l-alanine showed negative correlation with the *Mailhella* genus (r=-0.92 and -0.89, *P<0.01*) respectively. Epelsiban concentration was also negatively correlated with the *Paeniclostridium* genus (r= -0.89, *P<0.01*) (**Figure 9**).

## 4 Discussion

### 4.1 Effect of rumen acidosis on the gut microbiota in calves

Under normal physiological conditions, the gut is maintained in a relatively stable state by the close attachment of physiologically dominant anaerobic bacteria to the intestinal mucosa, forming a gut barrier (Chen et al., 2022). However, when calves experience rumen acidosis, the fermentation pattern in the abomasum changes, thereby affecting the stability and diversity of the rumen microbiota (Faniyi et al., 2019).

Alpha diversity analysis of the gut microbiota revealed a sample coverage index of over 99%, indicating that the sample size was sufficient to accurately reflect the types and diversity of gut microbiota in calves with rumen acidosis. In addition, the diversity index (Sob index) and abundance index (Chao index and ACE index) in the rumen acidosis group were significantly higher than those in the healthy group, indicating an increased proportion of harmful bacterial genera or the proliferation of other harmful bacteria in the calves gut due to rumen acidosis, leading to increase the diversity of gut microbiota. This finding was consistent with the description of J. C. Plaizier et al (Plaizier et al., 2018). In this study, a total of 10 phyla and 28 dominant bacterial genera were detected in the rumen acidosis group. At the phylum level, the dominant phyla in both the rumen acidosis group and the healthy group were Firmicutes and Bacteroidetes. The Proteobacteria phylum was significantly lower in calves with rumen acidosis compared to the healthy group, while the Desulfobacterota phylum was significantly higher. The relative abundance of Firmicutes showed an increasing trend in the rumen acidosis group, while the relative abundance of Bacteroidetes showed a decreasing trend. This finding contradicted the results of a study conducted by K. Wen et al., indicating that rumen acidosis significantly reduced the abundance of Proteobacteria, one of its signature phyla, and greatly disrupted the diversity of normal gut microbiota (Wen et al., 2021). Furthermore, the study also demonstrated that subacute rumen acidosis increased the relative abundance of Firmicutes in calves, which was consistent with the findings of the aforementioned study (Fu et al., 2022). Another study showed that rumen acidosis increased the relative abundance of Firmicutes and decreased the relative abundance of Bacteroidetes (Nagata et al., 2018). These studies indicated that the rumen acidosis indeed altered the normal gut microbiota in calves.

At the genus level, there were significant differences in the composition and proportion of gut microbial genera between the two groups. *Prevotella* and *Succinivibrio* were dominant genera (Li et al., 2022). In this study, the abundance and relative abundance of *Prevotella* (Ji et al., 2023), *Muribaculaceae*, *Succinivibrio* (Connors et al., 2018), *Prevotella* (Lourenco et al., 2020), *Agathobacter*, and *Parabacteroides* (Zafar and Saier, 2021) were significantly lower in the rumen acidosis group compared to the healthy group of calves, while the abundance and relative abundance of *Christensenellaceae_R-7* and *Parabacteroides* were significantly higher. *Prevotella* is a protein-degrading bacterium in the rumen and intestinal mucosa of ruminants, mainly degrading the semi-fiber components of food and promoting the degradation of non-fiber polysaccharides and pectin (Pang et al., 2022). Rumen acidosis leads to the change in the gut microbiota in calves, resulting in the decreased feed efficiency. *Succinivibrio* is a dominant genus in the Proteobacteria phylum (Tapio et al., 2017), which produces succinic acid through hydrogen utilization and plays an important role. Not only does this compete with hydrogenotrophic methanogens for substrates, but succinate is a precursor for propionate production (McCabe et al., 2015; Bailoni et al., 2021), and most propionate in ruminants is produced via the succinate pathway. Ramayo-Caldas et al. found that *Succinivibrionaceae* could improve feed efficiency, reduced methane emission, and increased the propionate concentration in Holstein cows (Ramayo-Caldas et al., 2020). *Christensenellaceae_R-7* belongs to the Firmicutes phylum (Waters and Ley, 2019). Combined with the analysis at the phylum level, it could be inferred that the relative abundance of the Firmicutes phylum in the gut microbiota of calves with rumen acidosis showed an increasing trend, further indicating that rumen acidosis in calves altered the abundance of gut microbiota. LDA analysis results of the gut microbiota in calves with rumen acidosis revealed that it could affect the normal gut microbiota community in calves, promote the rapid growth and proliferation of harmful bacterial communities and inhibit the proliferation of beneficial bacterial communities, result in an imbalance of the normal microbial flora.

### 4.2 Effect of rumen acidosis of calves on serum metabolites

Non-targeted metabolomics is a high-throughput data-driven approach widely used in the study of inflammation-related metabolic markers and pathways, providing a theoretical basis for the diagnosis and appropriate treatment of inflammation-related diseases. In this study, the non-targeted metabolomics based on LC-MS/MS platform combined with multivariate data statistical analysis methods were used to analyze the serum of calves in the rumen acidosis and healthy groups. The study found that the differential metabolites associated with rumen acidosis of calves mainly included niacin, nicotinic acid, L-glutamine and carnosine. These differential metabolites were mainly enriched in the niacin and nicotinic acid metabolism pathway and the histidine metabolism pathway. Niacin is the main precursor of nicotinamide adenine dinucleotide (NAD^+^) and its involvement in the metabolic pathway affects the synthesis of related metabolites. Nicotinic acid, also known as vitamin B3, is converted to niacin through transamination in the body, and niacin is the main precursor of NAD^+^ (Penberthy, 2009; Zhai et al., 2009). In the synthesis of NAD^+^, nicotinamide phosphoribosyltransferase (NAMPT) is the rate-limiting enzyme in this reaction pathway. NAMPT catalyzes the transfer of the phosphoribosyl group from 5’-phosphoribosyl-1’-pyrophosphate to nicotinamide mononucleotide (NMN) and pyrophosphate. NMN is converted to NAD^+^ under the action of nicotinamide adenine dinucleotide transferase (Luk et al., 2008; Sampath et al., 2015). NAD+ acts as an activator of SIRT1 and activates SIRT1 by regulating the ratio of NAD^+^/NADH. SIRT1 is a positive regulator of NF-кB, which is considered as an important transcription factor involved in the production of pro-inflammatory cytokines (Imai, 2009; Matsushita et al., 2013; Revollo et al., 2007; Zha et al., 2023). The study by Yu Ma et al. have found that regulating niacin and nicotinic acid metabolism could achieve anti-inflammatory effects (Ma et al., 2016). Zhou et al. found that disruption of niacin and nicotinamide metabolism could lead to a series of adverse reactions caused by inflammation and oxidative stress induced by hypertension (Zhou et al., 2023). This study found that the levels of niacin and nicotinamide metabolites in the serum of calves with rumen acidosis were significantly lower than those in the healthy group, leading to a decrease in the biosynthesis of NAD^+^. The reduction in NAD^+^ synthesis inhibits the activation of SIRT1, thereby reducing the inhibition of the NF-кB pathway, leading to the release of inflammatory factors and ultimately rumen acidosis. In conclusion, niacin and nicotinamide inhibit the occurrence of inflammation in calves by regulating niacin and nicotinamide metabolism.

Carnosine is a dipeptide molecule (β-alanyl-L-histidine) with anti-inflammatory, antioxidant, anti-glycation, and chelating properties (Prakash et al., 2021). Zheng et al. found that the presence of carnosine could increase the secretion of IL-10, GM-CSF, and TNF-α in the body while reduce the secretion of IL-8 (Zheng et al., 1996). In addition, carnosine has the antioxidant and anti-aging properties, including better maintenance of muscle strength and pH buffering properties, playing an important role in the stability and anti-fatigue (Guiotto et al., 2005). According to previous studies, the metabolism of alanine, aspartic acid, and glutamic acid is related to carnosine metabolism and is regulated by carnosine metabolism (Sookoian and Pirola, 2012). In addition, certain metabolic processes such as pyruvate metabolism, β-alanine metabolism, histidine metabolism, pantothenic acid and coenzyme A biosynthesis are also related to carnosine metabolism (Ostfeld and Hoffman, 2023). This study found that the level of carnosine in the serum of calves with rumen acidosis was significantly lower than that in the normal group, indicating that rumen acidosis affected the normal amino acid metabolism in calves, thereby affecting the synthesis of carnosine. It also suggests that inflammation inhibits the anti-inflammatory effect of carnosine. In conclusion, carnosine can be used as one of the metabolic markers to determine rumen acidosis in calves.

Glutamine is the main fuel and biological precursor of mammalian intestinal cells (Reeds and Burrin, 2001), including ruminants such as cattle (Okine et al., 1995) and sheep (Beaulieu et al., 2001), and involved in maintaining intestinal mucosal integrity (Potsic et al., 2002) and inhibiting the activation of inflammatory cytokines (Kim and Kim, 2017). L-glutamine is usually used as a functional antioxidant and energy supplement in the body. It is converted to glutamate and ammonia through deamination in the mitochondria of the small intestine, providing energy for the small intestine (Wang et al., 2022). This study found that the level of L-glutamine in the serum of calves with rumen acidosis was significantly lower than that in the calves of the healthy group, indicating that rumen acidosis affected the normal mechanism of the calf intestine. The decrease in L-glutamine leads to a decrease in the fuel and biological precursor required for the calves energy metabolism, resulting in the insufficient energy supply and ammonia conversion. Rumen acidosis disrupts the metabolism in the abomasum, leading to a decrease in the level of L-glutamine, which in turn causes an imbalance in the intestinal ecology of calves. Therefore, the level of L-glutamine in serum can also be used as one of the metabolic markers for evaluating rumen acidosis in calves.

### 4.3 Correlation analysis between serum metabolites and gut microbiota in calves with rumen acidosis

In the rumen acidosis group, compared to the normal calves, there was a significant increase in the abundance of the *Christensenellaceae_R-7* genus. This increase in the genus could affect the normal physiological functions of calves, leading to disrupt their glycolytic function. Calves over-ferment glucose, producing excessive amounts of acetate and butyrate, thereby interfering with normal glucose metabolism pathways. It further confirmed that the association between rumen acidosis in calves and dysbiosis of the microbial community. Additionally, (3beta, 4beta, 15alpha, 16beta, 25s)-4, 8, 15, 16, 26-pentahydroxycholest-5-en-3-yl beta-d-xylopyranoside is associated with monosaccharide synthesis. In this study, we found a positive correlation between the concentration of (3beta, 4beta, 15alpha, 16beta, 25s)-4, 8, 15, 16, 26-pentahydroxycholest-5-en-3-yl beta-d-xylopyranoside in serum metabolites and the *Christensenellaceae_R-7* genus in the gut microbiota. This suggested that an increase in this metabolite promoted the proliferation of the *Christensenellaceae_R-7* genus. Furthermore, Gemfibrozil can inhibit 1-O-β-glucuronidation (Tornio et al., 2017). The study found a negative correlation between the concentration of Gemfibrozil and the *Christensenellaceae_R-7* genus. This indicated that the proliferation of the *Christensenellaceae_R-7* genus inhibited the production of Gemfibrozil metabolites in serum, thereby affecting the normal physiological function of this metabolite. In summary, through the correlation analysis of gut microbiota and serum metabolites in calves, rumen acidosis primarily affected the excessive fermentation of products in the body’s glucose metabolism pathway, leading to metabolic disorders and loss of regulation of normal pathways, resulting in the onset of disease in calves. Therefore, it could be proven that rumen acidosis not only altered the gut microbiota in calves but also intervened with the diversity and abundance of post-intestinal microbial communities by affecting normal metabolic reactions.

## 5 Conclusion

The occurrence of rumen acidosis can induce changes in the gut microbiota of calves, with a significant increase of the *Christensenellaceae_R-7* genus and a significant decrease of *Prevotella* and *Succinivibrio* genera. Additionally, the occurrence of rumen acidosis can also induce changes in the serum metabolites including niacin, niacinamide, L-glutamine, and carnosine.

## Data availability statement

The data of 16s DNA has been uploaded. ID PRJNA1126434 http://www.ncbi.nlm.nih.gov/bioproject/1126434 Metabolomic data is being uploaded.

## Ethics statement

The animal studies were approved by Animal Ethics Committee of Lanzhou Institute of Animal Husbandry and Veterinary Medicine, Chinese Academy of Agricultural Sciences. The studies were conducted in accordance with the local legislation and institutional requirements. Written informed consent was obtained from the owners for the participation of their animals in this study.

## Author contributions

FW: Conceptualization, Data curation, Methodology, Writing – original draft, Writing – review & editing, Formal analysis, Validation, Visualization. PJ: Visualization, Writing – review & editing, Formal analysis, Methodology. HY: Methodology, Writing – review & editing, Data curation, Formal analysis, Software, Validation. XZ: Project administration, Writing – review & editing, Conceptualization, Resources, Supervision. XW: Writing – review & editing, Conceptualization, Project administration, Resources, Supervision.

## References

[B1] Aditya S. Humer E. Pourazad P. Khiaosa-Ard R. Zebeli Q. (2018). Metabolic and stress responses in dairy cows fed a concentrate-rich diet and submitted to intramammary lipopolysaccharide challenge. Animal. 12, 741–749. doi: 10.1017/S1751731117002191 28893334

[B2] Bailoni L. Carraro L. Cardin M. Cardazzo B. (2021). Active rumen bacterial and protozoal communities revealed by RNA-based amplicon sequencing on dairy cows fed different diets at three physiological stages. Microorganisms. 9, 754. doi: 10.3390/microorganisms9040754 33918504 PMC8066057

[B3] Beaulieu A. D. Overton T. R. Drackley J. K. (2001). Substrate oxidation by isolated ovine enterocytes is increased by phlorizin-induced glucosuria. Can. J. Anim. Sci. 81, 585–588. doi: 10.4141/A01-032

[B4] Chen M. Lin W. Li N. Wang Q. Zhu S. Zeng A.. (2022). Therapeutic approaches to colorectal cancer via strategies based on modulation of gut microbiota. Front. Microbiol. 13. doi: 10.3389/fmicb.2022.945533 PMC9389535 35992678

[B5] Connors J. Dawe N. Van Limbergen J. (2018). The role of Succinate in the regulation of intestinal inflammation. Nutrients. 11, 25. doi: 10.3390/nu11010025 30583500 PMC6356305

[B6] Faniyi T. O. Adegbeye M. J. Elghandour M. M. M. Y. Pilego A. B. Salem A. Z. M. Olaniyi T. A.. (2019). Role of diverse fermentative factors towards microbial community shift in ruminants. J. Appl. Microbiol. 127, 2–11. doi: 10.1111/jam.14212 30694580

[B7] Fu Y. He Y. Xiang K. Zhao C. He Z. Qiu M.. (2022). The role of rumen microbiota and its metabolites in subacute ruminal acidosis (SARA)-induced inflammatory diseases of ruminants. Microorganisms. 10, 1495. doi: 10.3390/microorganisms10081495 35893553 PMC9332062

[B8] Gressley T. F. Hall M. B. Armentano L. E. (2011). Ruminant Nutrition Symposium: Productivity, digestion, and health responses to hindgut acidosis in ruminants. J. Anim. Sci. 89, 1120–1130. doi: 10.2527/jas.2010-3460 21415422

[B9] Guiotto A. Calderan A. Ruzza P. Borin G. (2005). Carnosine and carnosine-related antioxidants: a review. Curr. Med. Chem. 12, 2293–2315. doi: 10.2174/0929867054864796 16181134

[B10] Imai S. (2009). The NAD World: a new systemic regulatory network for metabolism and aging–Sirt1, systemic NAD biosynthesis, and their importance. Cell Biochem. Biophys. 53, 65–74. doi: 10.1007/s12013-008-9041-4 19130305 PMC2734380

[B11] Ji H. Tan D. Chen Y. Cheng Z. Zhao J. Lin M. (2023). Effects of different manganese sources on nutrient digestibility, fecal bacterial community, and mineral excretion of weaning dairy calves. Front. Microbiol. 14. doi: 10.3389/fmicb.2023.1163468 PMC10232960 37275150

[B12] Khafipour E. Krause D. O. Plaizier J. C. (2009). A grain-based subacute ruminal acidosis challenge causes translocation of lipopolysaccharide and triggers inflammation. J. Dairy Sci. 92, 1060–1070. doi: 10.3168/jds.2008-1389 19233799

[B13] Kim M. H. Kim H. (2017). The roles of Glutamine in the intestine and its implication in intestinal diseases. Int. J. Mol. Sci. 18, 1051. doi: 10.3390/ijms18051051 28498331 PMC5454963

[B14] Li J. Lian H. Zheng A. Zhang J. Dai P. Niu Y.. (2022). Effects of different roughages on growth performance, nutrient digestibility, ruminal fermentation, and microbial community in weaned Holstein calves. Front. Vet. Sci. 9. doi: 10.3389/fvets.2022.864320 PMC9315432 35903131

[B15] Lourenco J. M. Kieran T. J. Seidel D. S. Glenn T. C. Silveira M. F. D. Callaway T. R.. (2020). Comparison of the ruminal and fecal microbiotas in beef calves supplemented or not with concentrate. PloS One 15, e0231533. doi: 10.1371/journal.pone.0231533 32282837 PMC7153887

[B16] Luk T. Malam Z. Marshall J. C. (2008). Pre-B cell colony-enhancing factor (PBEF)/visfatin: a novel mediator of innate immunity. J. Leukoc. Biol. 83, 804–816. doi: 10.1189/jlb.0807581 18252866

[B17] Ma Y. Bao Y. Wang S. Li T. Chang X. Yang G.. (2016). Anti-inflammation effects and potential mechanism of Saikosaponins by regulating nicotinate and nicotinamide metabolism and Arachidonic acid metabolism. Inflammation. 39, 1453–1461. doi: 10.1007/s10753-016-0377-4 27251379

[B18] Mao S. Zhang R. Wang D. Zhu W. (2012). The diversity of the fecal bacterial community and its relationship with the concentration of volatile fatty acids in the feces during subacute rumen acidosis in dairy cows. BMC Vet. Res. 8, 237. doi: 10.1186/1746-6148-8-237 23217205 PMC3582618

[B19] Matsushita T. Sasaki H. Takayama K. Ishida K. Matsumoto T. Kubo S.. (2013). The overexpression of SIRT1 inhibited osteoarthritic gene expression changes induced by interleukin-1β in human chondrocytes. J. Orthop Res. 31, 531–537. doi: 10.1002/jor.22268 23143889

[B20] McCabe M. S. Cormican P. Keogh K. O’Connor A. O’Hara E. Palladino R. A.. (2015). Illumina MiSeq phylogenetic amplicon sequencing shows a large reduction of an uncharacterised Succinivibrionaceae and an increase of the methanobrevibacter Gottschalkii clade in feed restricted cattle. PloS One 10, e0133234. doi: 10.1371/journal.pone.0133234 26226343 PMC4520551

[B21] Nagata R. Kim Y. H. Ohkubo A. Kushibiki S. Ichijo T. Sato S. (2018). Effects of repeated subacute ruminal acidosis challenges on the adaptation of the rumen bacterial community in Holstein bulls. J. Dairy Sci. 101, 4424–4436. doi: 10.3168/jds.2017-13859 29477528

[B22] Niederwerder M. C. (2018). Fecal microbiota transplantation as a tool to treat and reduce susceptibility to disease in animals. Vet. Immunol. Immunopathol. 206, 65–72. doi: 10.1016/j.vetimm.2018.11.002 30502914 PMC7173282

[B23] Oetzel G. R. (2017). Diagnosis and management of subacute ruminal acidosis in dairy herds. Vet. Clin. North Am. Food Anim. Pract. 33, 463–480. doi: 10.1016/j.cvfa.2017.06.004 28847417

[B24] Okine E. K. Glimm D. R. Thompson J. R. Kennelly J. J. (1995). Influence of stage of lactation on glucose and glutamine metabolism in isolated enterocytes from dairy cattle. Metabolism. 44, 325–331. doi: 10.1016/0026-0495(95)90162-0 7885277

[B25] Ostfeld I. Hoffman J. R. (2023). The effect of β-Alanine supplementation on performance, cognitive function and resiliency in soldiers. Nutrients. 15, 1039. doi: 10.3390/nu15041039 36839397 PMC9961614

[B26] Pang K. Dai D. Yang Y. Wang X. Liu S. Huang W.. (2022). Effects of high concentrate rations on ruminal fermentation and microbiota of yaks. Front. Microbiol. 13. doi: 10.3389/fmicb.2022.957152 PMC9558216 36246255

[B27] Penberthy W. T. (2009). Nicotinamide adenine dinucleotide biology and disease. Curr. Pharm. Des. 15, 1–2. doi: 10.2174/138161209787185779 19149596

[B28] Plaizier J. C. Danesh, Mesgaran. M. Derakhshani H. Golder H. Khafipour E. Kleen J. L.. (2018). Review: Enhancing gastrointestinal health in dairy cows. Animal 12, s399–s418. doi: 10.1017/S1751731118001921 30139397

[B29] Potsic B. Holliday N. Lewis P. Samuelson D. DeMarco V. Neu J. (2002). Glutamine supplementation and deprivation: effect on artificially reared rat small intestinal morphology. Pediatr. Res. 52, 430–436. doi: 10.1203/00006450-200209000-00021 12193680

[B30] Prakash M. D. Fraser S. Boer J. C. Plebanski M. de Courten B. Apostolopoulos V. (2021). Anti-cancer effects of Carnosine-A dipeptide molecule. Molecules. 26, 1644. doi: 10.3390/molecules26061644 33809496 PMC8002160

[B31] Ramayo-Caldas Y. Zingaretti L. Popova M. Estellé J. Bernard A. Pons N.. (2020). Identification of rumen microbial biomarkers linked to methane emission in Holstein dairy cows. J. Anim. Breed Genet. 137, 49–59. doi: 10.1111/jbg.12427 31418488 PMC6972549

[B32] Reeds P. J. Burrin D. G. (2001). Glutamine and the bowel. J. Nutr. 131, 2505S–8S,discussion 2523S-4S. doi: 10.1093/jn/131.9.2505S 11533302

[B33] Revollo J. R. Grimm A. A. Imai S. (2007). The regulation of nicotinamide adenine dinucleotide biosynthesis by Nampt/PBEF/visfatin in mammals. Curr. Opin. Gastroenterol. 23, 164–170. doi: 10.1097/MOG.0b013e32801b3c8f 17268245

[B34] Sampath D. Zabka T. S. Misner D. L. O’Brien T. Dragovich P. S. (2015). Inhibition of nicotinamide phosphoribosyltransferase (NAMPT) as a therapeutic strategy in cancer. Pharmacol. Ther. 151, 16–31. doi: 10.1016/j.pharmthera.2015.02.004 25709099

[B35] Sookoian S. Pirola C. J. (2012). Alanine and aspartate aminotransferase and glutamine-cycling pathway: their roles in pathogenesis of metabolic syndrome. World J. Gastroenterol. 18, 3775–3781. doi: 10.3748/wjg.v18.i29.3775 22876026 PMC3413046

[B36] Tang R. Yang W. Song J. Xiang K. Li S. Zhao C.. (2024). The rumen microbiota contributed to the development of mastitis induced by subclinical ketosis. Microb. Pathog. 187, 106509. doi: 10.1016/j.micpath.2023.106509 38185451

[B37] Tapio I. Snelling T. J. Strozzi F. Wallace R. J. (2017). The ruminal microbiome associated with methane emissions from ruminant livestock. J. Anim. Sci. Biotechnol. 8, 7. doi: 10.1186/s40104-017-0141-0 28123698 PMC5244708

[B38] Tornio A. Neuvonen P. J. Niemi M. Backman J. T. (2017). Role of gemfibrozil as an inhibitor of CYP2C8 and membrane transporters. Expert Opin. Drug Metab. Toxicol. 13, 83–95. doi: 10.1080/17425255.2016.1227791 27548563

[B39] Trevisi E. Riva F. Filipe J. F. S. Massara M. Minuti A. Bani P.. (2018). Innate immune responses to metabolic stress can be detected in rumen fluids. Res. Vet. Sci. 117, 65–73. doi: 10.1016/j.rvsc.2017.11.008 29179031

[B40] Wang S. Wang F. Kong F. Cao Z. Wang W. Yang H.. (2022). Effect of supplementing different levels of L-Glutamine on holstein calves during weaning. Antioxidants. 11, 542. doi: 10.3390/antiox11030542 35326192 PMC8944981

[B41] Waters J. L. Ley R. E. (2019). The human gut bacteria Christensenellaceae are widespread, heritable, and associated with health. BMC Biol. 17, 83. doi: 10.1186/s12915-019-0699-4 31660948 PMC6819567

[B42] Welch C. B. Ryman V. E. Pringle T. D. Lourenco J. M. (2022). Utilizing the gastrointestinal microbiota to modulate cattle health through the microbiome-gut-organ axes. Microorganisms. 10, 1391. doi: 10.3390/microorganisms10071391 35889109 PMC9324549

[B43] Wen K. Zhao M. M. Liu L. Khogali M. K. Geng T. Y. Wang H. R.. (2021). Thiamine modulates intestinal morphological structure and microbiota under subacute ruminal acidosis induced by a high-concentrate diet in Saanen goats. Animal. 15, 100370. doi: 10.1016/j.animal.2021.100370 34583314

[B44] Zafar H. Saier M. (2021). Gut Bacteroides species in health and disease. Gut Microbes 13, 1–20. doi: 10.1080/19490976.2020.1848158 PMC7872030 33535896

[B45] Zebeli Q. Metzler-Zebeli B. U. (2012). Interplay between rumen digestive disorders and diet-induced inflammation in dairy cattle. Res. Vet. Sci. 93, 1099–1108. doi: 10.1016/j.rvsc.2012.02.004 22370295

[B46] Zha D. Yang Y. Huang X. Wang Z. Lin H. Yang L.. (2023). Nicaraven protects against endotoxemia-induced inflammation and organ injury through modulation of AMPK/Sirt1 signalling in macrophages. Eur. J. Pharmacol. 946, 175666. doi: 10.1016/j.ejphar.2023.175666 36944380

[B47] Zhai R. G. Rizzi M. Garavaglia S. (2009). Nicotinamide/nicotinic acid mononucleotide adenylyltransferase, new insights into an ancient enzyme. Cell Mol. Life Sci. 66, 2805–2818. doi: 10.1007/s00018-009-0047-x 19448972 PMC11115848

[B48] Zheng L. M. Ojcius D. M. Garaud F. Roth C. Maxwell E. Li Z.. (1996). Interleukin-10 inhibits tumor metastasis through an NK cell-dependent mechanism. J. Exp. Med. 184, 579–584. doi: 10.1084/jem.184.2.579 8760811 PMC2192723

[B49] Zhou Z. Chen J. Cui Y. Zhao R. Wang H. Yu R.. (2023). Antihypertensive activity of different components of Veratrum alkaloids through metabonomic data analysis. Phytomedicine. 120, 155033. doi: 10.1016/j.phymed.2023.155033 37647672

